# Supplementary material for: Genetic and Functional Characterization of Congenital HCMV Clinical Strains in Ex Vivo First Trimester Placental Model
Source: Pathogens. 2023 Jul 27;12(8):985. doi: 10.3390/pathogens12080985 (PMC10460061; doi:10.3390/pathogens12080985)
Supplement: Supplementary file 1 [file pathogens-12-00985-s001.zip › pathogens-2374632-supplementary.pdf]

Supplementary data

1.A

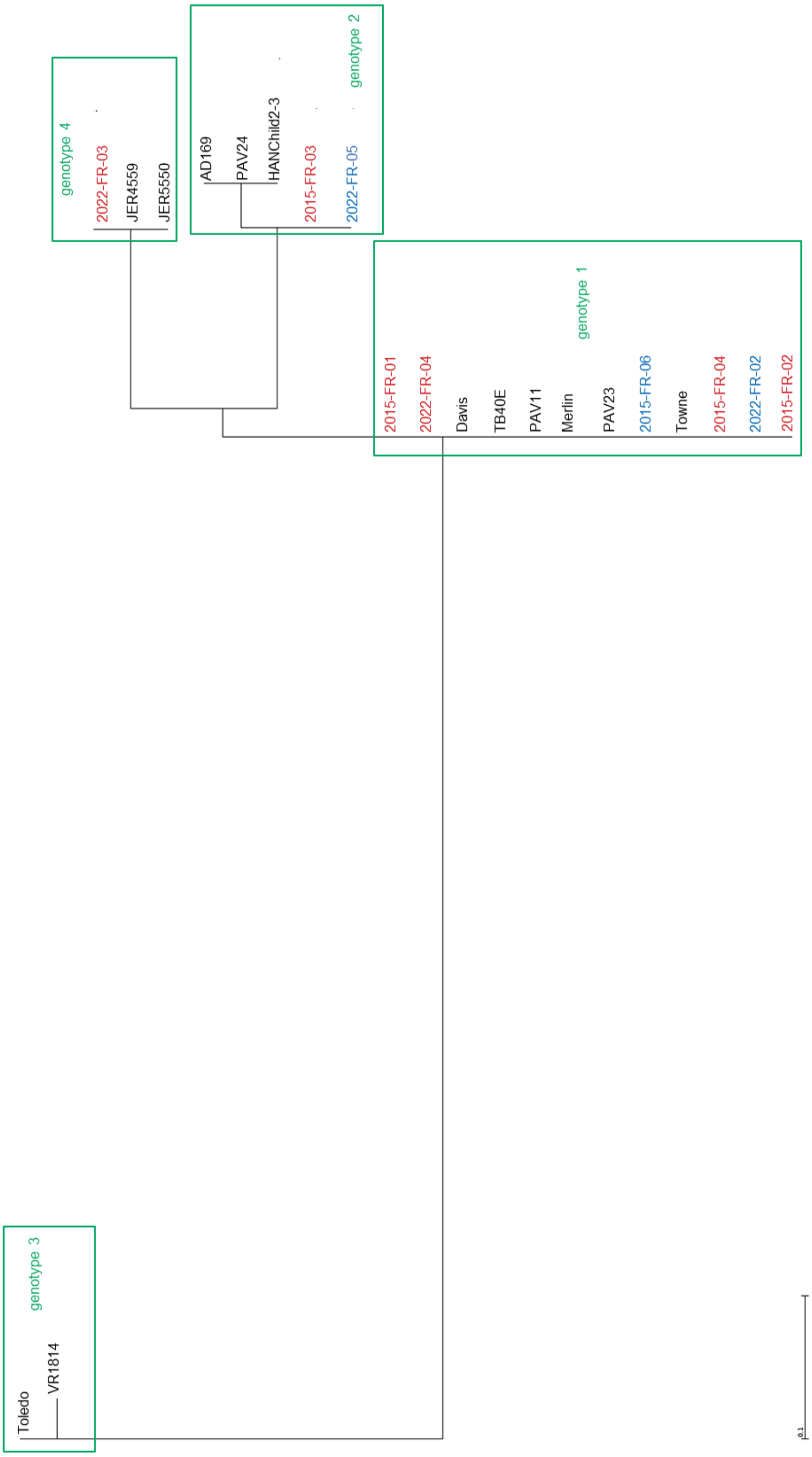

1.B

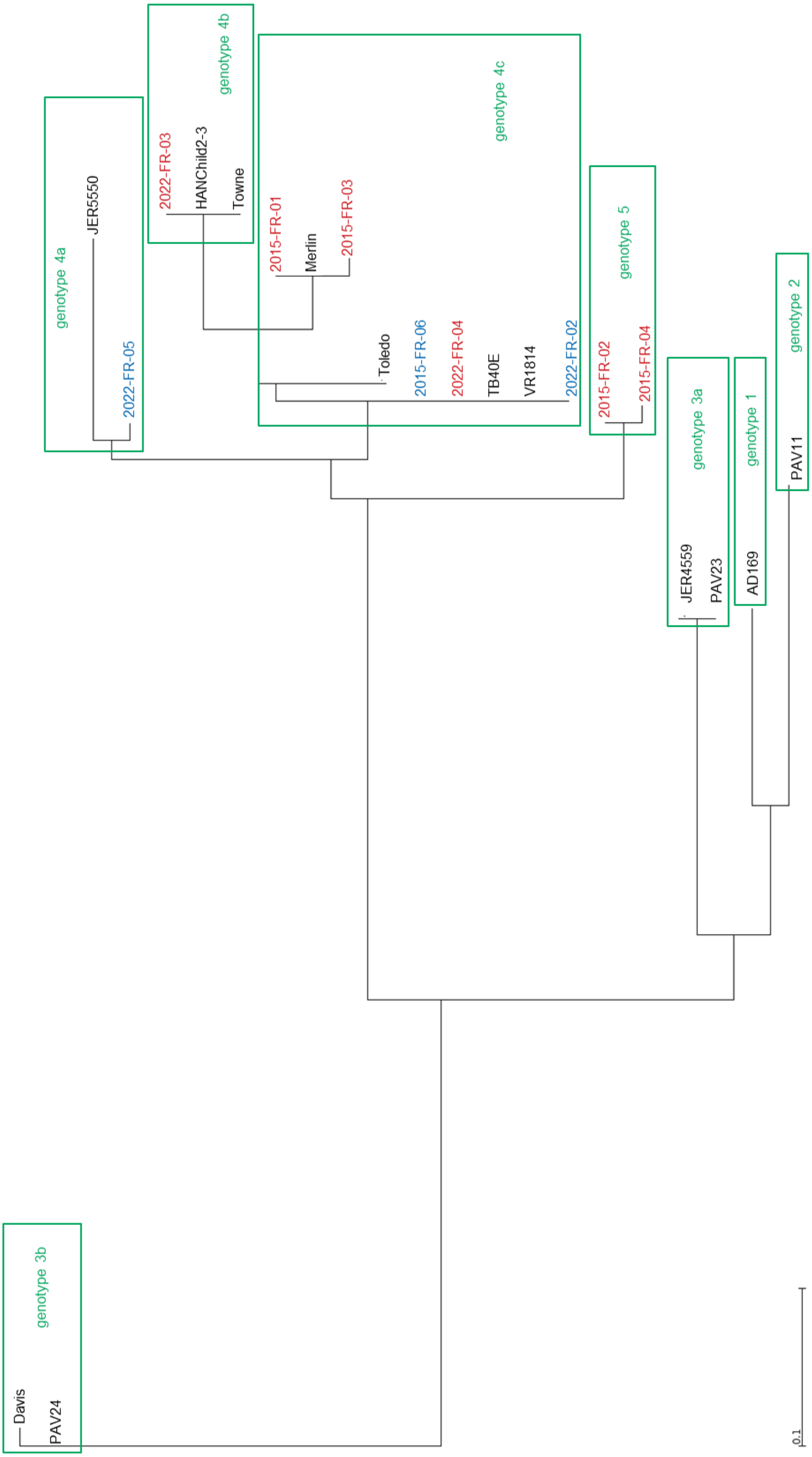

genotype 4a
JER5550
2022-FR-05
genotype 4b
2022-FR-03
HANChild2-3
Towne
genotype 4c
2015-FR-01
Merlin
2015-FR-03
Toledo
2015-FR-06
2022-FR-04
TB40E
VR1814
2022-FR-02
genotype 5
2015-FR-02
2015-FR-04
genotype 3a
JER4559
PAV23
genotype 1
AD169
genotype 2
PAV11
genotype 3b
Davis
PAV24
0.1

1.C

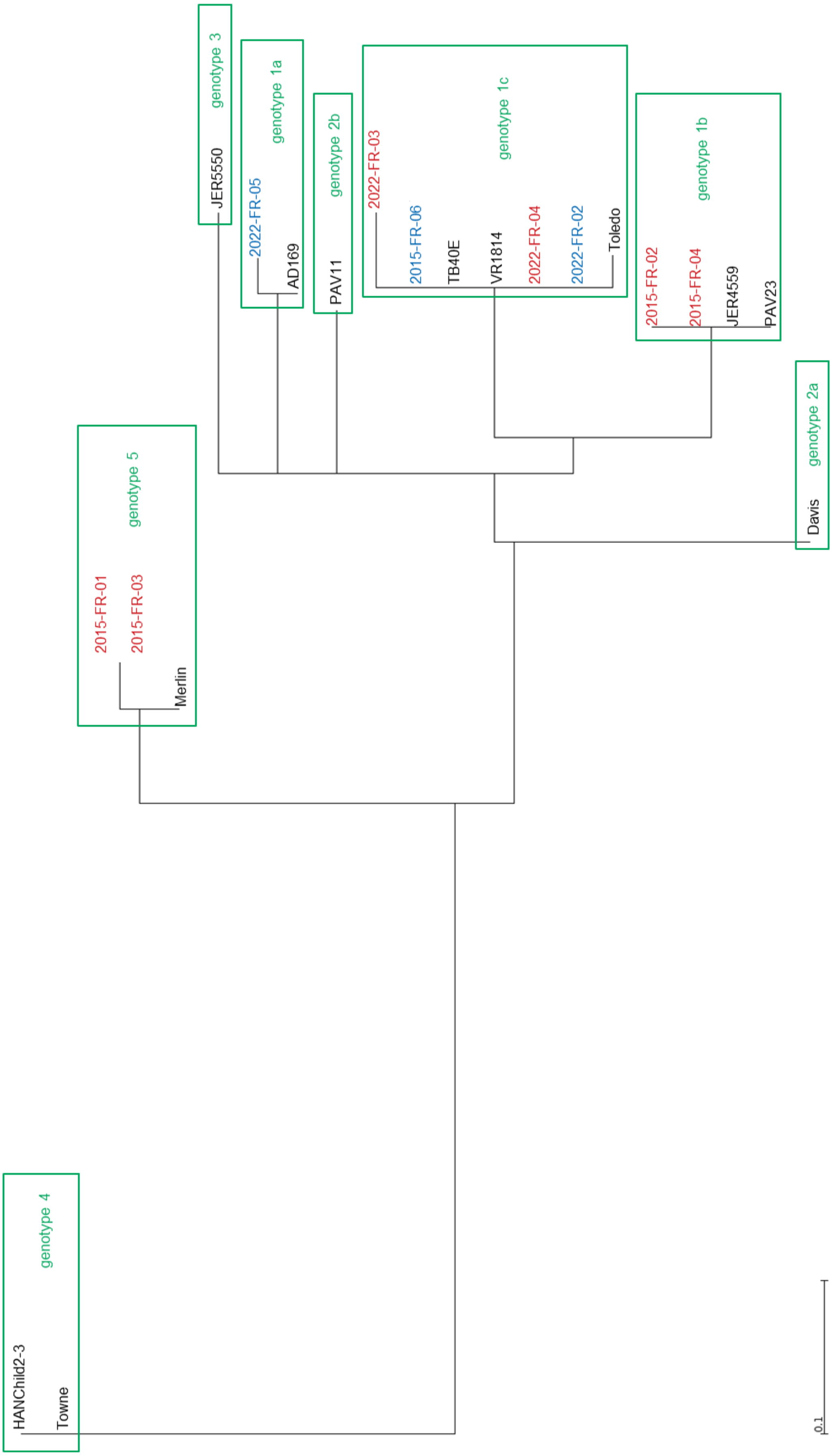

genotype 3
JER5550
genotype 1a
2022-FR-05
AD169
genotype 2b
PAV11
genotype 1c
2022-FR-03
2015-FR-06
TB40E
VR1814
2022-FR-04
2022-FR-02
Toledo
genotype 1b
2015-FR-02
2015-FR-04
JER4559
PAV23
genotype 2a
Davis
genotype 5
2015-FR-01
2015-FR-03
Merlin
genotype 4
HANChild2-3
Towne
0.1

1.D

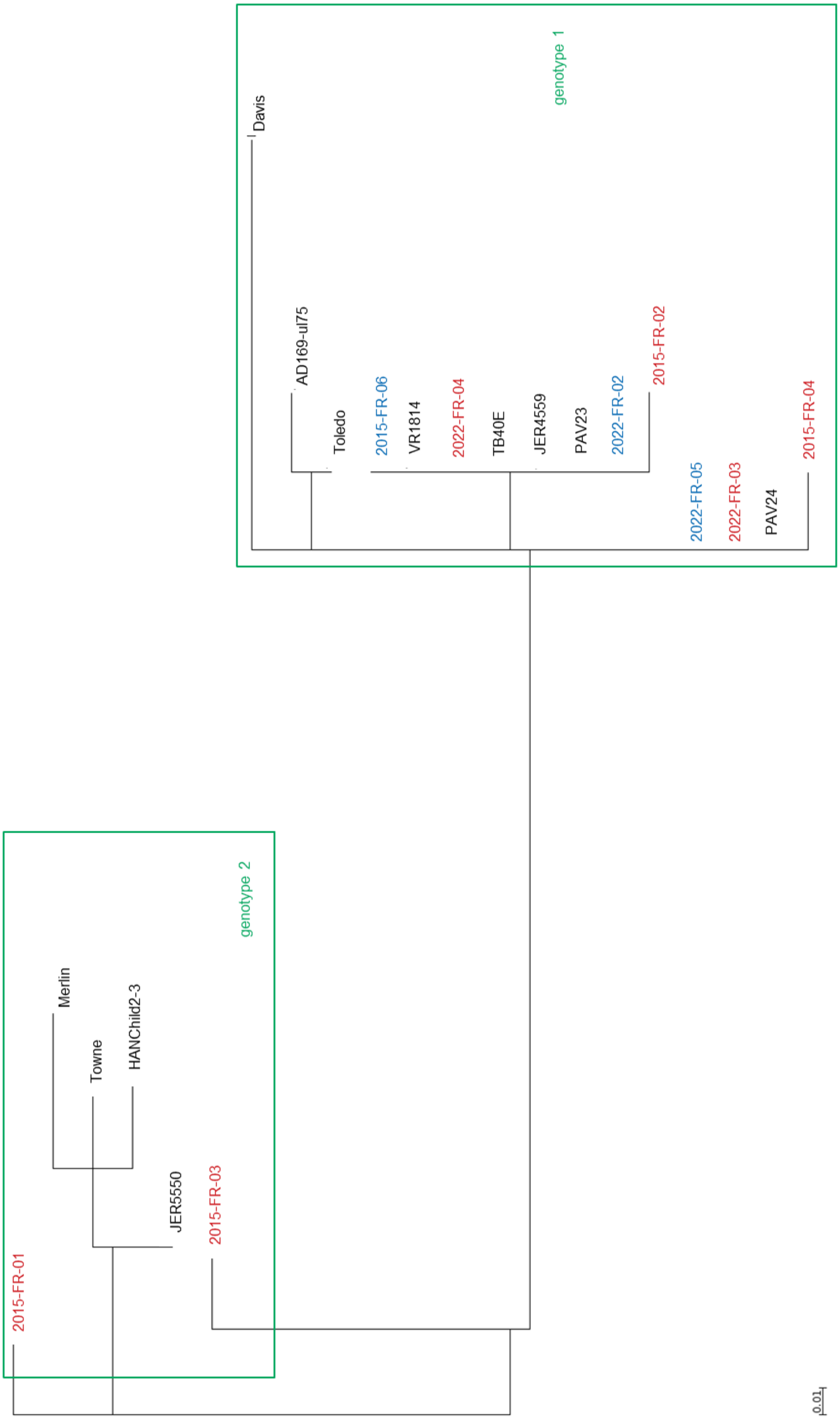
genotype 1
Davis
AD169-ul75
Toledo
2015-FR-06
VR1814
2022-FR-04
TB40E
JER4559
PAV23
2022-FR-02
2015-FR-02
2022-FR-05
2022-FR-03
PAV24
2015-FR-04
genotype 2
Merlin
Towne
HANChild2-3
JER5550
2015-FR-03
2015-FR-01
0.01

1.E

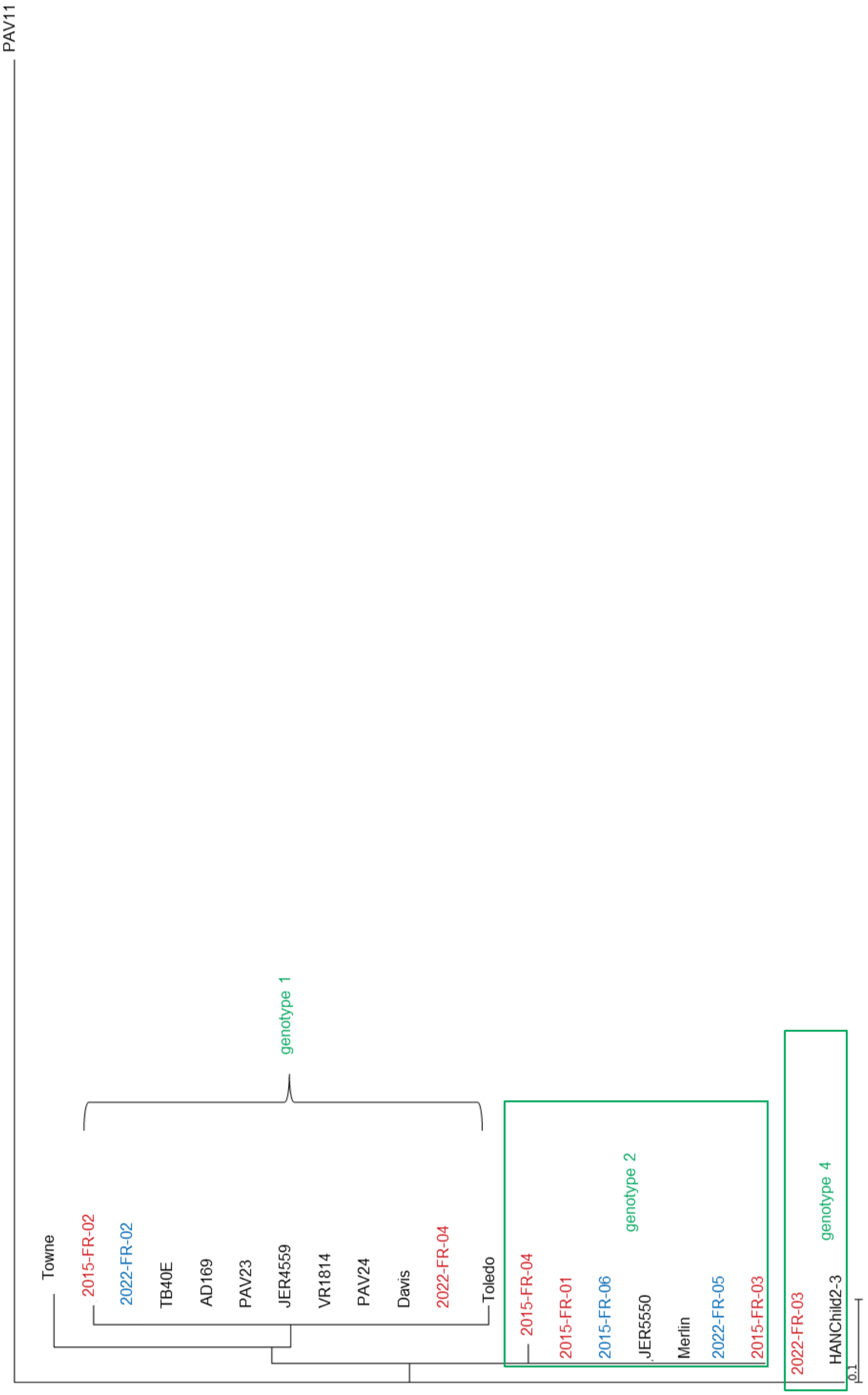

Figure 1. Phylogenetic trees of HCMV glycoproteins extracted amino acids sequences. Sequences were aligned using Muscle and trees were generated

with iqtree using 1000 bootstraps. Reference strains are in black, symptomatic strains in red, asymptomatic strains in blue. (A) Phylogenetic tree of *UL55* (gB) (B) Phylogenetic tree of *UL73* (gN) (C) Phylogenetic tree of *UL74* (gO) (D) Phylogenetic tree of *UL75* (gH) (E) Phylogenetic tree of *UL115* (gL)

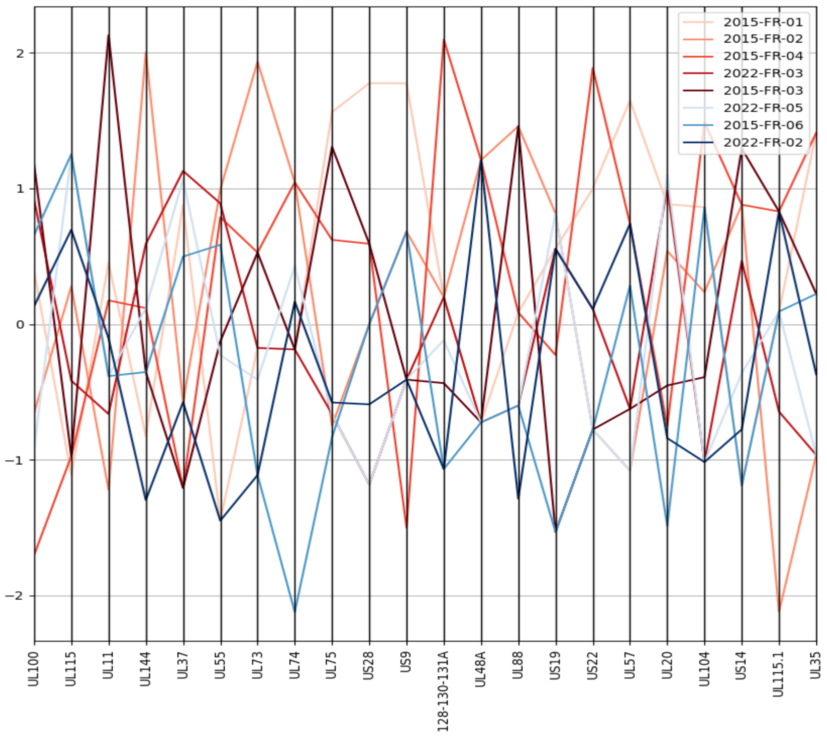

Figure 2. Parallel coordinate chart was obtained from normalized genomic variation counts and enabled to have another view of multivariate data presented in PCA. Analyzed genes are indicated in abscissa, and a normalized count of positions divergent from TB40/E genome is indicated in ordinate. Symptomatic strains are presented in red tone and asymptomatic in blue tones. We can have a better view of relationships between variables that are not apparent in PCA (UL74, US28, US9,…) as all analyzed genes are represented.

Table 1. **N**umber of non-synonymous mutations per gene and per clinical sample.

Gene	2015-FR-01	2015-FR-02	2015-FR-04	2015-FR-06	2022-FR-02	2022-FR-03	2022-FR-05	2015-FR-03
UL100	10	6	2	11	9	12	5	13
UL115	5	15	6	22	18	10	22	6
UL11	8	2	7	5	6	4	5	14
UL144	7	13	9	8	6	10	9	8
UL37	46	23	13	40	23	50	49	13
UL55	6	30	28	26	6	29	18	19
UL73	8	17	11	4	4	8	7	11
UL74	29	43	43	7	33	29	36	29
UL75	29	2	18	1	4	3	3	26
US28	11	8	9	8	7	6	6	9
US9	4	3	1	3	2	2	2	2
UL128-130-131A	6	6	12	2	2	6	5	4
UL48A	0	1	1	0	1	0	0	0
UL88	3	5	3	2	1	2	2	5
US19	8	9	5	0	8	8	9	0
US22	6	4	7	4	5	5	4	4
UL57	7	1	5	4	5	2	1	2

UL20	57	49	19	2	17	60	62	26
UL104	6	5	7	6	3	3	3	4
US14	8	13	13	8	9	12	10	14
UL115	5	2	6	5	6	4	5	6
UL35	6	2	6	4	3	2	2	4
